# Costs and benefits of awns

**DOI:** 10.1093/jxb/erw140

**Published:** 2016-05-07

**Authors:** Zifeng Guo, Thorsten Schnurbusch

**Affiliations:** HEISENBERG-Research Group Plant Architecture, Leibniz Institute of Plant Genetics and Crop Plant Research (IPK), Corrensstr. 3, 06466 Stadt Seeland, OT Gatersleben, Germany

**Keywords:** Breeding, canopy temperature, drought, germplasm, harvest index, heritability, photosynthesis, screenings, test weight.

**Awns, which are derived from floral structures in grasses, are known to be critically important for photosynthesis and transpiration. However, arguments about the costs and benefits of their growth and development for crop grain yield are still ongoing. In this issue of *Journal of Experimental Botany* (pages 2573–2586), Rebetzke *et al*. take the debate forward, showing that wheat awns are associated with specific grain traits in different environments.**

Globally, the most common colours for bird-dispersed fleshy fruits are red or black (Janson, 1983). Different colours appear at lower frequencies, and in these cases the fruit are mainly spread by other frugivores, suggesting that fruit colours are adaptive in attracting birds (Duan *et al.*, 2014). Similarly, plant breeding has triggered the dispersal and performance of awns in many areas of cultivation. Adaptation has also resulted in the seeds of many wild grass species having large, barbed awns, which deter seed-eating animals and assist in seed dispersal; and in wild wheat species, such as wild emmer [*Triticum dicoccoides* (Körn. ex Asch. & Graebner) Schweinf.], the awns flex as humidity levels change, which can help bury the seeds (Elbaum *et al.*, 2007; Evangelista *et al.*, 2011; Mach, 2015). However, after thousands of years of human selection, our domesticated wheats (*T. durum* and *T. aestivum*) have shorter or non-existent awns to facilitate grain harvesting, handling and storage (Mach, 2015) (see also Box 1).

**Box 1. A spiky affair**

**Figure:**
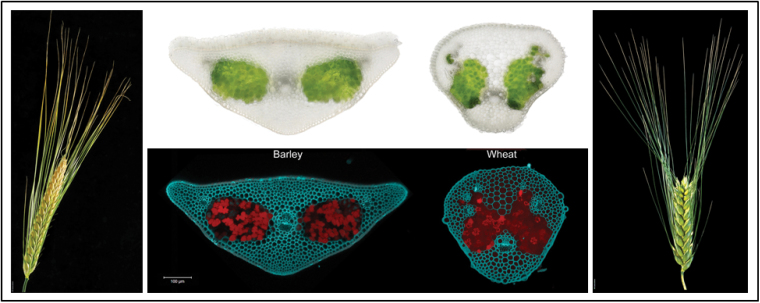
Awns represent an unusual floral expansion not seen in other major cereal crops, such as rice, maize or sorghum, but are a characteristic feature of barley (left) and wheat (right) spikes. Developmentally, awns are lemma-derived and photosynthetically active. Transverse sections exhibit anatomical features including the chlorophyll-containing cells.

Today, wheat is one of the most widely grown crops and an essential component of global food security – a substantial increase in yield is required to feed the increasing human population, which is predicted to rise to 9.74 billion by 2050 (World Population Prospects, 2015 Revision). Therefore, raising grain yield is still the main goal for wheat breeding, particularly as recent decades have seen a reduced rate of increase in wheat yields globally. Increasing assimilate partitioning into wheat’s grain-bearing structures, the spikes, is an important strategy for improving yield. The introduction of dwarfing Reduced height (Rht) genes greatly enhanced the partitioning of assimilates towards spikes (Rebetzke and Richards, 2000). However, current cultivars have already reached an optimum height, and alternative strategies need to be identified in order to achieve further yield increases.

Awns can be considered an alternative target for the improvement of wheat grain yield through their known functions, including photosynthesis, carbohydrate storage and increased water-use efficiency (Weyhrich *et al.*, 1995). Arguments about their putative role (costs or benefits) for grain yield are ongoing, although some functions have been validated. Considerable work in previous studies suggests that both positive and negative effects on wheat grain yield are possible with different growth conditions (Motzo and Giunta, 2002; Maydup *et al.*, 2010). Use of assimilates allowing awn development seems to be the main negative factor.

The research by Rebetzke *et al.* (2016) is particularly important in explaining the way in which awns affect grain yield. They found that awns are coupled with larger grain size and yield in less favourable environments but reduce grain number in more favourable environments. They also show that awns do not significantly affect the total number of spikelets and anthesis time, but instead markedly increase the number of sterile spikelets and grain size in some environments.

The most significant differences from previous work include the study of variable genetic backgrounds, a large number of environments, and a considerable number of traits: (1) four spring wheat populations with a range of diverse genetic backgrounds were developed containing multiple Near-Isogenic Line (NIL) pairs with variation for the presence and absence of full awns; (2) four separate sets of experiments representing a total of 25 environments (23 in Australia and two in Mexico) were conducted depending on the germplasm and environments sampled; and (3) for comparisons a number of important agronomic traits were measured in multiple years and environments while some traits were related to spike morphology. Separate glasshouse studies were also undertaken.

## Regulation of spike morphology by awns

Manipulating source–sink relations is an important strategy for wheat breeding. Spike morphology is a crucial phenotype for displaying the distribution of assimilates. Spike morphology-related traits (e.g. total number of spikelets; number of fertile spikelets; number of sterile spikelets; grain number per spikelet at apical, central and basal parts of spike; grain number and weight per spike; dry weight and photosynthetic surface area for spikelets) demonstrate great variation under variable growth conditions. Therefore, environmentally induced plasticity of spike morphology-related traits can in principle show the redistribution of assimilates.

Rebetzke *et al.* (2016) report that awns modify spike morphology, e.g. by increasing sterile spikelet number and grain size. In other words, assimilates are redistributed within the spike due to the allocation of assimilates to large and rapidly developing awns. We hypothesize that grain yield is influenced not only by the generation of the awn itself, but also by the redistribution of assimilates within the spike caused by awn development. This redistribution commences at around the time of terminal spikelet development and continues during stem elongation through to ear emergence. In addition, the awn-induced modification of spike morphology plays different roles (costs and benefits) for grain yield under variable environments, and this helps explain the different performances of grain yield in the ‘less and more favourable environments’ mentioned by Rebetzke *et al.* (2016). Increasing fruiting efficiency (i.e. number of grains set per unit spike dry weight at anthesis) is an important option for increasing grain yield without altering the allocation pattern of assimilates to the spike, as current cultivars already have an optimum plant height and it is not easy to increase assimilate partitioning towards the spike (Slafer *et al.*, 2015). Awn length demonstrates great variability and potential for modification across modern cultivars, so greater efforts are needed to understand more about the effects of awns on fruiting efficiency.

## Costs of awn setting

The influence of wheat awns on grain yield can be summarized as a balance between the costs of awn setting and the benefits of awn functions. As hypothesized by Rebetzke *et al.* (2016), allocation of assimilates to large and rapidly developing awns decreases fertile spikelet number and floret fertility, reducing grain number particularly in distal florets. Fertile florets are the outcome of floral development and demise, and floral demise occurs in a narrow time range and can be delayed by detillering (due to an increase of assimilate allocation) (Guo and Schnurbusch, 2015). Based on this, we hypothesize that the costs of awn setting will decrease assimilate partitioning to florets and may further trigger floral demise associated with fertile floret number. Moreover, it was previously shown that ovary size is associated with grain number and size (Guo *et al.*, 2015; Xie *et al.*, 2015). It is thus likely that awn development competes for assimilates during ovary growth and further influences grain yield.

Flowering time is commonly considered to be an important determinant for grain yield. Rebetzke *et al.* (2016) found that awns have no significant effects on time to anthesis but influence grain yield to some extent. It is highly likely that awns function on grain yield through their effects on the duration of specific sub-stages (e.g. time between green to yellow anther stage; Guo and Schnurbusch, 2015). Future studies are required to resolve this issue.
